# Clinical Characteristics of Children With SARS-CoV-2 Infection in a Hospital in Latin America

**DOI:** 10.3389/fped.2022.921880

**Published:** 2022-06-09

**Authors:** Laura F. Niño-Serna, Eliana López-Barón, Isabel Cristina Maya Ángel, Carolina Tamayo-Múnera

**Affiliations:** ^1^Department of Pediatrics, Hospital Pablo Tobón Uribe, Medellín, Colombia; ^2^Pediatric Critical Care Unit, Hospital Pablo Tobón Uribe, Medellín, Colombia

**Keywords:** pediatrics, comorbidity, inpatients, pediatric intensive care unit, Latin America, COVID-19

## Abstract

**Objective:**

COVID-19 infections have shown a different behavior in children than in adults. The objective of this study was to describe the clinical characteristics and severity of SARS-CoV-2 infection in pediatric patients seen at a reference hospital in Colombia.

**Method:**

A descriptive, observational study in patients under the age of 18 years with a positive test for SARS-CoV-2 infection (RT-PCR or antigen) between April 2020 and March 2021. Multiple variables were studied, including demographic data, clinical characteristics, lab measurements, treatments administered, intensive care unit admission, and mortality.

**Results:**

A total of 361 patients were included of whom 196 (54%) were males. The median age was 3 years. Of all the patients, 65 (18%) were asymptomatic. The majority of patients had no comorbidities (*n* = 225, 76%). In those who were symptomatic (*n* = 296, 82%), the most frequent complaints were fever (*n* = 178, 60%), nasal congestion (*n* = 164, 55%) and cough (*n* = 149, 50%). Chest x-rays were normal in 73 patients (50%). When abnormalities were found, interstitial (29%) and alveolar (12%) patterns were the most prevalent. One hundred and fifty-seven children (53%) required general ward hospitalization, and 24 patients (8%) required pediatric intensive care admission. The global mortality was 0.8% (3 patients).

**Conclusions:**

The majority of cases were asymptomatic or mild. However, a significant percentage of patients required general ward admission, and some even required intensive care. The main symptom of COVID-19 infections in newborns was apnea. A second COVID-19 RT-PCR may be necessary to detect infections in critically ill patients with a high clinical suspicion of the disease if an initial test was negative.

## Introduction

In December 2019, an outbreak of severe acute respiratory syndrome coronavirus infection was described in Wuhan, China. As of March 2020, a global pandemic had been declared. A wide array of disease manifestations has been observed, ranging from no symptoms at all to serious respiratory distress and even death. Adults, especially the elderly, have suffered the greatest impact of this disease (1). At the time of writing, the World Health Organization (WHO) had confirmed over 500 million cases worldwide, with almost 6 million deaths attributed directly to COVID-19 infection (2). In Latin America, around 56 million cases and more than 1.2 million deaths have been reported (3). In Colombia, an upper-middle-income country, around 6 million cases and more than 100,000 deaths had been reported as of February 2022 (4). Of all the positive cases in Colombia up to February 2022, 11% were reported in people under the age of 19 (5).

The reported pediatric COVID-19-related deaths have been higher in low and middle-income countries (91.5%) compared to high-income countries (8.5%), with an excess of deaths in Latin America (6). In South America, there are economic, social, and health disparities. Previous studies have shown that certain traits of poor countries have a direct impact on COVID-19 outcomes. This includes low socioeconomic status, overcrowding, higher use of public transportation, absence of potable water, and informal work (7). As a result of the worst scenarios experimented on by infected adults, healthcare services, including pediatric ones, were rearranged to meet the increasing requirements of that age group (8). This came to destabilize vulnerable health systems in which lack of government support, excessive centralization, inequities, and inadequate access were already historical. As a consequence, the pandemic became a major challenge (9).

As the pandemic has progressed, several papers have provided a better understanding of the severe acute respiratory syndrome coronavirus 2 (SARS-CoV-2). This includes a glimpse into its pathophysiology, immune response, treatments, as well as the spectrum of manifestations in the pediatric population, in whom the disease has been less severe. Previous studies carried out in countries with limited resources are mainly focused on the characteristics of the multisystem inflammatory syndrome in children (MIS-C) due to its particular specificity for this age group (10–13). Therefore, case series not related to MIS-C are lacking.

The objective of this study was to describe the clinical and imaging characteristics of the infection, the use of specific diagnostic tests and the severity of the SARS-CoV-2 infection in patients admitted to a reference pediatric hospital in Colombia, an upper-middle-income country in Latin America.

## Methods

A descriptive, observational study was carried out at a tertiary care hospital in Medellín, Colombia. The Hospital is a high complexity reference center, with 78 pediatric hospital beds and 27 pediatric and neonatal intensive care beds. Around 928 patients per month (11,139 during the study period) were seen in the emergency department and 2,928 were admitted during the study period.

### Population

Children under 18 years of age who had a positive test for SARS-CoV-2 [real-time reverse transcription-polymerase chain reaction (RT-PCR) or COVID-19 antigen] and were treated in one of our hospital services: ambulatory care, pediatric emergency room (ER), hospitalization ward, or pediatric intensive care unit (PICU) between April 2020 and March 2021 were included.

### COVID-Protocol Attention in the Institution

The institutional protocol for diagnosing SARS-CoV-2 infection was based on RT-PCR. Indications for testing included at least one of the following: respiratory distress, odynophagia, asthenia, anosmia, hypogeusia, runny nose, or fever. If a positive result was available, then no further workup was carried out. In hospitalized patients, if the initial test was negative, then a second RT-PCR test 48–72 h was done. Patients transferred from other institutions were also tested if a previous test was negative; no mattered if was either antigen or RT-PCR. Also, RT-PCR was customary in patients who had surgery or as part of a stem cell transplantation protocol.

### Data Collection

The medical charts were reviewed and the data was registered on an Excel form previously designed for this purpose with the following variables: age, sex; clinical variables such as the onset of symptoms, presenting symptoms of the disease, epidemiological contact, comorbidities, area of care (hospital ward, ER, PICU, ambulatory care); complications like multisystem inflammatory syndrome in children (MIS-C), PICU admission, and respiratory failure; lab and diagnostic test results; treatment received during hospitalization (pharmacological treatment, mechanical ventilation, vasopressor support, dialysis); length of hospital and PICU stay; and outcome at discharge: death or recovery.

### Statistical Analysis

A descriptive analysis was performed. Qualitative variables are presented as frequencies and proportions. For quantitative variables, normality was assessed using the Shapiro-Wilk test and they are reported as median or mean with interquartile range (IQR) or standard deviation (SD). The data were processed on SPSS version 20 (SPSS Inc. Chicago, IL, USA). The study was approved by the institution's ethics committee and did not require informed consent because of the national statements.

## Results

### Demographic and Epidemiological Characteristics

**Figure 1:**
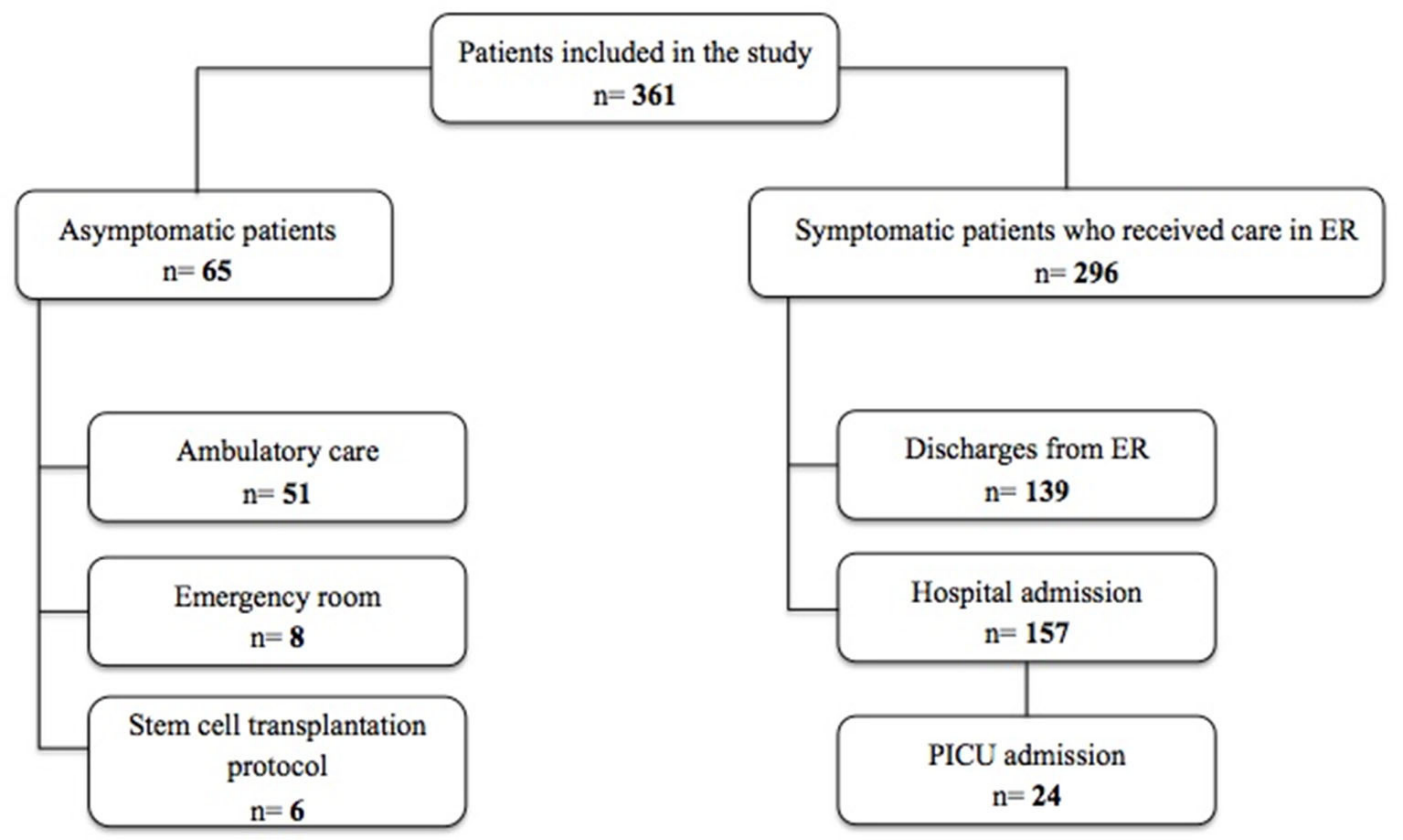
Flowchart of the included patients.

A total of 361 patients were included. The median age was 3 years (IQR 1–10 years). Seven patients were newborns (2%). The demographic characteristics of all the patients are reported in Table 1. In Figure 1 we present a flowchart of the included patients.

**Table 1:** Demographic characteristics of all the patients.

**Variable**	***n* (%)**
**Age**	
<1 year	82 (23)
1–5 years	135 (37)
6–11 years	71 (20)
12–17 years	73 (20)
Female sex	165 (46)
**Place of origin**	
Antioquia	347 (96)
Major cities	315 (91)
Peripheral towns	32 (9)
Chocó	4 (1)
Colombian Caribbean region	4 (1)
Others	6 (2)

*Peripheral town: is a town that does not have a health infrastructure for pediatric care*.

### Clinical Characteristics

Out of all the patients, 65 (18%) were asymptomatic. The vast majority were tested because of positive close contact, 51(79%) were outpatients and eight (12%) received attention in the ER. Also, six patients (9%) underwent testing as a part of the stem cell transplantation protocol.

The bulk of the sample, 269 (82%), was composed of symptomatic patients, and all received pediatric medical care. Of these, 157 (53%) patients were hospitalized. Most of the children admitted to ER were 2 years old or younger (*n* = 159, 52%); the youngest was 7 days old and the oldest was 17 years old. The longest time that elapsed between the onset of symptoms and consultation was 20 days, in one patient. Table 2 shows the characteristics of the symptomatic patients.

**Table 2:** Characteristics of patients with COVID-19 symptoms.

**Variable**	***N* = 296**
Time elapsed from the onset of symptoms to	3 days (1–4)
consultation, median (IQR)
**Principal diagnosis on admission**, ***n*** **(%)**	
Upper-respiratory infection due to COVID-19	176 (60)
Lower-respiratory infection due to COVID-19	33 (11)
Bronchiolitis	16 (48)
Pneumonia	17 (52)
Acute gastroenteritis	18 (6)
Asthma	15 (5)
Neurological (epilepsy, seizures, hydrocephaly)	10 (4)
Sepsis	9 (3)
Urinary tract infection	7 (2)
MIS-C	4 (1)
Others^a^	24 (8)
**Symptoms or signs present on admissions**, ***n*** **(%)**
Fever	178 (60)
Rhinorrhea/nasal congestion	164 (55)
Cough	149 (50)
General malaise	75 (25)
Gastrointestinal symptoms	74 (25)
Respiratory distress syndrome	42 (14)
Odynophagia	32 (11)
Headache	24 (8)
Stridor	11 (4)
Rash	9 (3)
Hypoxemia	8 (3)
Seizure	6 (2)
Apnea	5 (2)
Ageusia	5 (2)
Anosmia	4 (1)
**Comorbidities**, ***n*** **(%)**
None	225 (76)
Asthma	25 (8)
Chronic lung disease	15 (5)
Immunosuppression / immunodeficiency	14 (5)
Hemato-oncological disease	9 (3)
Others^b^	8 (3)
**Chest x-ray**, ***n*** **(%)**	*N* = 145
Normal	73 (50)
Interstitial pattern	42 (29)
Alveolar opacity	17 (12)
Atelectasis	8 (6)
Others^c^	5 (3)
**Positive first test**, ***n*** **(%)**	271 (91)
PCR	267 (99)
Antigen	4 (1)
Positive second test (RT-PCR), *n* (%)	27 (9)
Positive IgM serology, *n* (%)	1 (0.3)
Complications, *n* (%)	17 (6)
**Coinfection**, ***n*** **(%)**	44 (15)
Viral	20 (48)
Bacterial	22 (52)
Hospitalization, *n* (%)	157 (53)
Admission to intensive care, *n* (%)	24 (8)
Hospital stay, median (IQR)	2 days (1–3)

*IgM, immunoglobulin M; MIS-C, Multi-system inflammatory syndrome in children. ^a^ Others: BRUE, trauma, arterial hypertension, appendicitis, bone marrow aplasia, acute myeloid leukemia, bacteremia, tachycardia, malnutrition, cystic fibrosis, mononucleosis, syncope, sickle cell anemia, sexual abuse, osteomyelitis. ^b^ Others: pulmonary hypertension 3 patients, arterial hypertension 2 patients, obesity 2 patients, biological therapy 1 patient. ^c^ Others: hyperinflation 3 patients, pleural effusion 1 patient, ground glass 1 patient*.

The main diagnosis on admission was directly related to SARS-CoV-2 infection in 213 patients (72%) and included: upper-respiratory infection (common cold, pharyngitis, sinusitis, or laryngitis), lower-respiratory infection (bronchiolitis and pneumonia) and MIS-C. In 83 patients (28%) the reason for admission was another non-COVID-related diagnosis and a test was performed as a part of the institutional protocol. The most frequent symptoms were fever in 60%, rhinorrhea in 55%, and cough in 50% of the cases (Table 2). Of all the patients treated, 9 (3%) had MIS-C and were treated with immunoglobulin infusions and high-dose steroids. Another form of presentation was apnea, characteristic of newborns (*n* = 4/5, 80%). None of these patients had other viruses detected, like respiratory syncytial virus (RSV) or influenza. If asymptomatic cases and those with mild respiratory infections are grouped, they add up to 204 (57%).

SARS-CoV-2 infection was diagnosed with an initial positive test in 271 cases (92%); the remaining 25 patients (8%) required a second RT-PCR test to confirm the diagnosis. Of those initially negative, 23 were RT-PCR and 2 were antigen tests.

Coinfections were detected in 42 patients (14%); 20 children (48%) had an associated viral infection, 9 (45%) due to RSV, 5 (25%) due to influenza; 4 (20%) due to both, RSV and influenza. One patient had cytomegalovirus infection, and another had Epstein-Barr virus. Twenty-two patients (52%) had bacterial coinfections. The isolated microorganisms were *E. coli, E. cloacae, P. mirabilis, Staphylococcus aureus, S. epidermidis, Salmonella, S. agalactiae, E. faecalis, Pseudomonas aeruginosa, Clostridium difficile* and *Candida parapsilosis*. *Mycoplasma pneumoniae* was found in 5 of 28 (18%) patients.

Table 3 describes the results of laboratory tests. A C-reactive protein (CRP) >10 mg/dl was found in 28/169 patients (16%), neutropenia was reported in 30/180 patients (16%) and moderate thrombocytopenia (<100,000) was found in 12 patients (7%).

**Table 3:** Laboratory tests performed on patients with COVID-19 infection.

**Variable**	***n***	
PCR (mg/dL)^a^	169	1 (0.2–4.2)
ESR (mm/hour)^a^	24	37.5 (18.2–63.7)
Leukocyte count (mm^3^)^a^	180	9,200 (6,400–12,500)
Absolute neutrophils (mm^3^)^a^	180	5,038 (2,400–8,046)
Hemoglobin (g/dL)^a^	181	12.6 (11.8–13.5)
Platelets (mm^3^)^a^	180	302,000 (238,250–371,500)
D-dimer (ng/mL)^a^	13	2,633 (1,284–11,732)
LDH (U/l)^a^	17	299 (226–398)
Ferritin (ng/mL)^a^	14	527 (169–1,091)
Fibrinogen (mg/dL)^b^	14	617 (±263)

^a^*median and interquartile range; ^b^mean and standard deviation*.

Two patients (0.5%) were suspected of having healthcare-associated COVID-19 infection after 14 days of hospitalization for a different cause, one for aplastic anemia and another undergoing bone marrow transplantation due to leukemia.

The global mortality for the entire cohort was 0.8% (3 patients). Two patients died of septic shock and multiple organ dysfunction: a healthy 4-year-old with *Staphylococcus aureus* infection and a newborn with suspected immunodeficiency with *Candida parapsilosis* infection. The third child, a 5-year-old with cerebral palsy and epileptic encephalopathy, was receiving palliative care and died of respiratory failure. In this case, no other etiological agent other than SARS-CoV-2 was identified.

### Intensive Care

Of the 296 symptomatic patients, 24 (8%) were admitted to the PICU or the neonatal care unit with a positive test for COVID-19. Only in six patients (25%), the cause of admission was related to COVID-19. The median length of stay in intensive care was 4 days (IQR 3–6 days). Of the patients that were admitted to the PICU, seven (29%) had a false negative initial test, five RT-PCR tests, and two antigen tests. Table 4 describes the characteristics of the patients admitted to the PICU.

**Table 4:** Characteristics of patients admitted to the pediatric intensive care unit.

**Variable**	**No (%)**
**Age**, ***n*** **(%)**
<1 year	10 (42)
1–5 years	4 (16)
6–11 years	5 (21)
12–17 years	5 (21)
Female sex, *n* (%)	12 (50)
Time elapsed between the onset of	3 days (1.2–5)
symptoms and consultation, median (IQR)
Coinfection, *n* (%)	10 (42)
**Principal diagnosis on admission**, ***n*** **(%)**
Sepsis	8 (35)
Acute COVID-19 respiratory infection	5 (22)
BRUE	4 (17)
Hypertensive urgency/emergency	2 (9)
Asthma	1 (4)
Status epilepticus and respiratory failure	1 (4)
Urinary tract infection	1 (4)
MIS-C	1 (4)
**Comorbidities**, ***n*** **(%)**
None	13 (54)
Chronic neuropathy	6 (25)
Immunodeficiency	2 (8)
Asthma	1 (4)
Hemato-oncological disease	1 (4)
Arterial hypertension	1 (4)
Chest x-ray, *n* (%)	*N* = 22
Normal	3 (14)
Interstitial pattern	11 (50)
Alveolar opacity	4 (18)
Atelectasis	4 (18)

*IQR, interquartile range; BRUE, Brief resolved unexplained events; MIS-C, Multi-system inflammatory syndrome in children*.

The treatment received in the intensive care unit was as follows: invasive or non-invasive mechanical ventilation in 9 patients (38%), including two patients who required prone positioning, with a median duration of 3 days (IQR 1.5–16 days). Three patients required a high flow cannula. Seven (78%) patients who required mechanical ventilation had bacterial coinfection. Four patients (15%) received vasopressor support, and three patients (11%) received renal replacement therapy. Chest x-rays characteristics of those admitted to the intensive care unit are reported in Table 4.

## Discussion

This study describes the demographic, diagnostic, clinical, and imaging characteristics of 361 patients under the age of 18 years diagnosed with SARS-CoV-2 infection at a tertiary care hospital in an upper-middle-income Latin American country. The majority of cases were either symptomatic or mild. We found a highly variable clinical and radiological presentation, perhaps a reflection of the diverse geographical and economical background of the population studied. Additionally, the diagnostic protocol carried out in our institution was successful in detecting false-negative cases, thus possibly preventing in-hospital infections.

The burden placed on health services during the COVID-19 pandemic substantially affected the care of pediatric patients. Given the fact of higher complications and mortality rates, infrastructure and human resources were redirected towards adults' attention. Evidence suggests that children suffered a drop in quality and delays in healthcare access (14). Moreover, as described by Kitano et al. (6) these differences may have been accentuated in low and middle-income countries, which further deepened the gap between nations concerning diagnosis, progression, and follow-up. This disparity is exemplified in mortality rates 35 times higher than those seen in high-income countries (0.43 vs. 0.012). Furthermore, there is an inverse relationship between PICU admissions and income so that access to these units is truncated in countries with limited resources (6).

Latin America is an example of the negative effects that lack of resources and a faulty healthcare structure had on the outcomes of this global emergency. Before the pandemic, most Latin American countries did not have the bare minimum recommended facilities to take care of patients as recommended by the WHO: at least 2.9 hospital beds per 1,000 inhabitants. For instance, Colombia and Peru had 1.6 and Ecuador 1.5. Similarly, only Argentina and Brazil met the essential critical care bed requirement of 6 per 100,000 inhabitants. Moreover, qualified human resources for critically ill patients in Latin America are scarce (9). Therefore, when available means were heavily shifted towards adult care, the already deficient pediatric infrastructure suffered a shortage of hospital and PICU beds. Our institution, aware of the situation caused by the pandemic, had to reduce pediatric critical care beds by 26% and general hospital beds by 22%. In 2020, there was a considerable reduction in pediatric admissions to hospitalization wards and pediatric intensive care units. A phenomenon registered throughout Latin America, mainly evidenced by lower admissions due to lower respiratory tract infections (15).

No less important were the effects of the pandemic on the global health of children and the socioeconomic factors that influence them. In the years 2020-2021 in Colombia, there was a notable decrease in morbidity and mortality due to respiratory diseases. On the other hand, child malnutrition increased significantly after lockdowns were lifted, evidencing low employment rates and high rates of informal work in the region and the country (9, 16). In this series, only 4% of patients lived in peripheral rural areas even if infection rates were similar to those of cities. This is a reflection of the limited access those populations have to the country's health system.

A notable difference from other studies was the prevalence between age groups. While in other studies, the infection was more prevalent in children over 6 years old, in this series we observed more cases in infants under 2 years old (52%). This age distribution could be explained by a selection bias in which parents of younger children seek medical attention in the ER worried about potentially worst outcomes and because the access to ambulatory care is difficult.

Although this study was carried out in a tertiary care center, the occurrence of comorbidities was low and 76% of patients did not have any comorbidity. Data from other studies are inconsistent in this regard. Li et al. (17) results are aligned with our findings whereas others report a greater proportion of comorbidities, for instance, studies from Peru (42%), Argentina (53.9%), and Brazil (38.9%) (18–20).

Every study reviewed, and ours, show that COVID-19 symptoms are similar to and indistinguishable from those of other common viral diseases affecting preschoolers and school-age children and comprise: fever, respiratory and gastrointestinal complaints (18, 19, 21–23). In this series, most patients suffered from acute upper respiratory infection, with a few cases of pneumonia and hypoxemia. Also, of all the patients admitted to the PICU, only 25% had a diagnosis directly related to COVID-19, unlike the clinical presentation in adults (24, 25).

In these series, the majority of patients had mild symptoms or were asymptomatic; just 6% had pneumonia and 5% had bronchiolitis, which explains the lower frequency of hypoxemia at the moment of ER admission. A similar trend was found by Rodriguéz-Portilla et al. (18). For many reasons and hypotheses, the severe COVID-19-pneumonia experienced by adults is very rare in children (26).

The largest Colombian study was carried out by Bolaños-Almeida (27). The records of around fifty thousand patients under 18 years with COVID-19 were reviewed. Hospital admissions only represented around 2.8% of cases, whereas PICU admissions were even lower, with <1% requiring intensive care, similar to another population-based study in Chile, where hospitalization in patients with COVID-19 was 2% (28). These data are not comparable to our results given the type of studies, but they give us a broader picture of the behavior of the disease in the pediatric population. Half of our patients required admission. This high figure could be explained by the nature of our institution and the complexity of our patients, as well as our center's protocols for care and follow-up. Ferraro et al. (19) in a single-center study in Argentina, found even higher admissions rates with a tally of 74.8%.

The performance of the diagnostic tests for COVID-19 infection, either antigen or RT-PCR, seems to be variable. Specifically, sensitivity is greatly influenced by the time elapsed since the onset of symptoms and the nasal swabbing (29). Moreover, significant false-negative rates and discordance between different RT-PCR have been described, although not in children (30, 31). In our case, 8% of tests were initially negative and this was even higher in PICU patients (29%). We believe that this is not negligible because COVID-19 infections are significantly contagious and entail confinements in affected patients. Thus, we hypothesize that a second test may be necessary to rule out or confirm the infection in critically ill patients with high clinical suspicion of COVID-19.

Hospital-acquired COVID-19 infections were rare (0.5%) and at a rate lower than previously estimated. For instance, Hendler et al. (20) found a frequency of 7.9% of nosocomial infections. We strongly believe that our diagnostic strategy contributed to these numbers. Since symptomatic patients were required to have two negative RT-PCRs for COVID-19, 48–72 h apart, for isolation measures to be lifted, the probability of hospital contagions was significantly lowered.

We found that 3% of patients suffered from MIS-C. Similar results have been reported in the series from Australia (1.3%) and Argentina (4.6%). On the other hand, a Peruvian study found higher rates of severe disease with 16.8% affected with MIS-C (18, 19, 32). Compared with other studies, our PICU admission rate (8%) was similar to that described by Ferraro et al. (18–20, 32, 33) (6.8%) and is rather average. PICU admissions figures range from 0.5 up to 28%.

A striking finding was that previously healthy patients were the ones mainly admitted to the PICU, although this has also been described in previous studies (10, 18). Besides, SARS-CoV-2 severe respiratory disease is rare in children when compared to adults and in our cohort was present in only 9 patients, 77% of whom had previous ailments.

A common complaint in neonates was apnea (80%), which prompted admission to the neonatal intensive care unit. These patients did not have a viral co-infection that frequently causes this symptom. This granted a greater risk in this population whit confirmed COVID-19 infection, a finding not described in other series (34). Regarding respiratory coinfection, the study by Wu et al. (35) reported similar germs but in different proportions; Mycoplasma infection being the most frequent in that series and RSV in ours.

Deaths were rare with an estimate of 0.8% and resembled those of most studies (19, 27, 36, 37). Patients with chronic illnesses such as pulmonary disease, immunodeficiencies, congenital heart disease, or neurological disease are more susceptible to death (22, 23, 38). Higher mortality rates have been reported and probably are explained by healthcare access constraints in the studied populations (18, 20).

Positive imaging findings were scarce in our series and general x-rays did not disclose abnormalities, except for patients in the PICU where pathological findings were the norm. Studies that have evaluated radiological variables differ somehow. Investigations carried out in Latin America and Spain found that peribronchial thickening, ground-glass opacities, consolidation, and vascular thickening were frequent (39, 40).

Patients were treated with immunomodulators, steroids and immunoglobulin, when severe infection or MIS-C ensued. In those cases with a diagnosis of asthma exacerbation, steroids were used. There are no other approved treatments for SARS-CoV-2 in Colombia.

An important limitation of this study is that being retrospective, we were sometimes unable to retrieve information for some variables. In addition, the study was conducted at a local and national reference center, which allows for selection bias, especially for critically ill patients.

In summary, this study provides insights into COVID-19 infection in children cared for at a high complexity hospital. By and large, the disease is mostly mild. However, a substantial number of patients required hospital or PICU admission. Therefore, specific arrangements should be implemented in these settings to confront the pandemic.

Finally, we found that, after an initially negative result, a second COVID-19 RT-PCR may be necessary to detect the infection in critically ill patients with a high clinical suspicion of the disease. This strategy may lower the rate of nosocomial infections.

## Data Availability Statement

The raw data supporting the conclusions of this article will be made available by the authors, without undue reservation.

## Ethics Statement

The studies involving human participants were reviewed and approved by Comité de Investigaciones y ética en Investigaciones del Hospital Pablo Tobón Uribe (Research and Research Ethics Committee of Pablo Tobón Uribe Hospital). Written informed consent from the participants' legal guardian/next of kin was not required to participate in this study in accordance with the national legislation and the institutional requirements.

## Author Contributions

LN-S conceptualized and designed the study, performed the data collection, the statistical analyses, drafted the initial manuscript, and reviewed the final manuscript. CT-M conceptualized and designed the study, performed the data collection, drafted the initial manuscript, and reviewed the final manuscript. IM and EL-B performed the data collection, drafted the initial manuscript, and reviewed the final manuscript. All authors approved the final manuscript as submitted and agree to be accountable for all aspects of the work.

## Conflict of Interest

The authors declare that the research was conducted in the absence of any commercial or financial relationships that could be construed as a potential conflict of interest.

## Publisher's Note

All claims expressed in this article are solely those of the authors and do not necessarily represent those of their affiliated organizations, or those of the publisher, the editors and the reviewers. Any product that may be evaluated in this article, or claim that may be made by its manufacturer, is not guaranteed or endorsed by the publisher.
